# Template method synthesis of mesoporous carbon spheres and its applications as supercapacitors

**DOI:** 10.1186/1556-276X-7-269

**Published:** 2012-05-29

**Authors:** Karolina Wilgosz, Xuecheng Chen, Krzysztof Kierzek, Jacek Machnikowski, Ryszard J Kalenczuk, Ewa Mijowska

**Affiliations:** 1 Institute of Chemical and Environment Engineering, West Pomeranian University of Technology, Szczecin, ul. Pulaskiego 10, Szczecin, 70-322, Poland; 2 Department of Polymer and Carbonaceous Materials, Wroclaw University of Technology, ul. Gdanska 7/9, Wroclaw, 50344, Poland

**Keywords:** Mesoporous silica, Carbon spheres, Mesoporous carbon

## Abstract

Mesoporous carbon spheres (MCS) have been fabricated from structured mesoporous silica sphere using chemical vapor deposition (CVD) with ethylene as a carbon feedstock. The mesoporous carbon spheres have a high specific surface area of 666.8 m^2^/g and good electrochemical properties. The mechanism of formation mesoporous carbon spheres (carbon spheres) is investigated. The important thing is a surfactant hexadecyl trimethyl ammonium bromide (CTAB), which accelerates the process of carbon deposition. An additional advantage of this surfactant is an increase the yield of product. These mesoporous carbon spheres, which have good electrochemical properties is suitable for supercapacitors.

## Background

Supercapacitors are a fast-developing technology in electric energy storage including lithium-ion secondary batteries, fuel cells, and electrical double-layer capacitors [1,2]. They are electrochemical storage devices able to fill the gap that exists between batteries and dielectric capacitors from the energy and power density point of view [3]. In recent years electric double layer capacitors (EDLCs) have evoked wide interest, because of their capability to supply high power in short-term pulse, which makes them very good energy storage devices for applications such as hybrid power sources for electrical vehicles, portable electronic devices, and pulse laser techniques [4]. Materials for EDLCs should have a huge surface area to accumulate large amount of charges and size-controllable porous channel system for the easy access to the electrolyte [5]. The most promising materials are carbon spheres because of their commercial availability, price, and abundance [6]. Mesoporous carbon has got remarkable properties such as high specific surface area, large pore volume, low density, thermal conductivity, electrical conductivity, good chemical and mechanical stability, and great application potential to catalysts, electrodes, batteries, sensors, adsorbents in separation processes, gas storage materials, and templates for fabricating nanostructures [7-10]. Mesoporous carbon spheres can be fabricated with the mesoporous silica spheres as the templates [11]. Production of mesoporous carbon spheres, which is an inverse replica templated from mesoporous silica, attracted increasing attention [12].

In this paper, a simple chemical vapor deposition method for mesoporous carbon spheres (sphere shape) fabrication is presented. This technique is different from the previously reported methods using mesoporous silica spheres [11,12] because they involve of carbon source being incorporated into the channels of mesoporous structures and subsequent carbonization process. These procedures require additional step to decompose surfactant and subsequent filling carbon into the mesoporous silica. In our case a single CVD process is needed to produce the carbon filled mesoporous silica spheres. These obtained mesoporous carbon spheres show good electrochemical properties in supercapacitors at high current load.

## Methods

### Synthesis of mesoporous silica spheres

m-SiO_2_ nanospheres were prepared as follows: surfactant hexadecyl trimethyl ammonium bromide (CTAB) (300 mg) was added to a mixture of ethanol (EtOH) (60 ml), distilled water (80 ml) and ammonia (25 wt %, 1,1 mL), then sonicated and subsequent vigorous stirred. After stirring for 30 min. tetraethyl orthosilicate (TEOS) (0,4 ml) was added to the reaction mixture and subsequently stirred at room temperature for 12 h. Finally, the product was obtained through centrifugation and dried.

### Synthesis of carbon filled m-SiO_2__C

The dried m-SiO_2_ treated by CTAB was used as a template to prepare the carbon spheres using CVD. This compositon was placed in an alumina boat and set in a tube furnace. Argon and ethylene were introduced at a flow rate of 100 sccm and 30 sccm, respectively. The temperature was raised to 800°C. and the process took 4 h. Afterwards, the resulting m-SiO_2__C spheres were thoroughly washed with hydrofluoric acid to remove the silica and obtain the final product.

### Characterization

X-ray diffraction (XRD) was conducted on a Philips diffractometer using Cu K_α_ radiation. Transmission electron microscopy (TEM) together with Energy Dispersive X-Ray spectrometer as its mode has been utilized to examine the dimensions, structural details and chemical composition of the samples (Tecnai F30 with a field emission gun operating at 200 kV). Raman scattering was conducted on a Renishaw micro Raman spectrometer (λ = 785 nm). The specific surface area was calculated by the Brunauer-Emmett-Teller (BET) metod via Micromeritics ASAP 2010 M instrument. The pore size distribution was determined using the Barret–Joner–Halenda (BJH) method. Thermogravimetric analysis (TGA) was carried out on 10 mg samples using the DTA-Q600 SDT TA Instrument at a heating rate of 10°C/min from room temperature to 900°C under air.

### Electrochemical measurement

Two-electrode capacitor of diameter 10 mm and mass of 9–10 mg were pressed from a mixture of active material (85%), polyvinylidene fluoride PVDF (10%) and acetylene black (5%). Electrode was composed of the mesoporous carbon spherical spheres and were separated by the glassy fibrous paper and placed between gold current collectors in a teflon Swagelok® type system where 1 mol l^–1^ H_2_SO_4_ solution was an electrolyte. Voltammetry experiments at a scan rate from 1 to 100 m V s ^-1^ and galvanostatic charge/discharge at a current density from 0.2 to 20 A g−^1^ were used for the estimation of the specific capacitance C expressed in farads (F) per gram of carbon spheres. In this work the results of voltammetry experiments for a better legibility were presented as a function of C = f (E). The VMP3 (Bio-logic Science Instruments, France) multichannel generators were used for the realization of the measurements.

## Results and discussion

**Figure 1:**
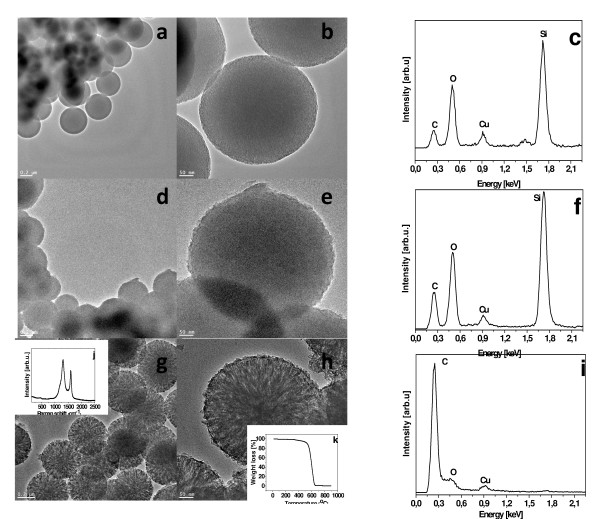
**TEM images.** Images of the mesoporous silica spheres with CTAB and correspondind EDX spectrum (**a**-**c**); mesoporous silica with deposited carbon and correspondind EDX spectrum (**d**-**f**), carbon spheres and correspondind EDX spectrum (**g**-**i**); Raman spectra of the carbon spheres (**j**); TGA profile of the carbon spheres (**k**)

CTAB was trapped in the silica channels, which has accelerated deposition of carbon during CVD. The mesoporous silica spheres with CTAB trapped inside, were placed in a horizontal quartz reactor and CVD reaction has started. After this reaction the composition was treated by HF and carbon spheres were obtained. TEM images of the structure of the mesoporous silica spheres (a-b), mesoporous silica with carbon (d-e) and carbon spheres (g-h) are shown in Figure 1. The morphology of the mesoporous silica spheres with CTAB in the channels used as the template is presented in (Figure 1a, b). They exhibit spherical morphology with a mean diameter of 410 nm and a size distribution of 40 nm. EDX reveals that next to Si and O a carbon signal is present (from CTAB composition) (Figure 1c). Cu comes from TEM grid. The mesoporous silica spheres with CTAB were treated in CVD reactor and the morphology of the samples is presented in Figure 1d, e. Here, one can see that the spherical structure of the samples changed the mean diameter up to 510 nm meaning that the carbon was deposited in the interstitial channels of the silica mesoporous and on its surface. The chemical composition of the material is enriched by the strong peak coming from deposited carbon (Figuer 1f).

Finally, the HF treatment of the samples resulted in preparation of mesoporous carbon spheres with channels radially oriented with respect to the sphere centre and the mean diameter of 410 nm (Figure 1g, h). It means that the carbon deposited on the spheres surface has been also removed successfully during the acid treatment. EDX spectrum confirm the removal of SiO_2_ (Figure 1i). To further analyse the final product the Raman spectrum was detected (see inset of Figure 1g). It clearly reveals two the tangential modes i.e. the G mode (1598 cm^−1^) that is derived from the graphite-like in-plane mode and the disorder induced D band (1300 cm^−1^). Additionally, TGA of the carbon spheres shown in the inset of Figure 1h indicates that the material starts to decompose in air at 526 ^0^ C. The weight loss increases rapidly with the further increase of the combustion temperature. This process lasts untill all of the carbon spheres were burnt off at about 650°C and at the final stage no ashes were present in the TGA cuvette after the thermal treatment. This clearly indicates the sample quality and the efficiency of silica removal upon HF treatment.

**Figure 2:**
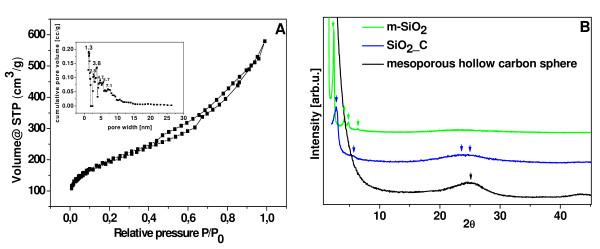
**Nitrogen adsorption isotherms and pore volume distribution functions.** For mesoporous carbon spheres (**a**); powder X-ray diffraction patterns of mesoporous hollow carbon spheres (**b**)

The sample was also measured by N_2_ adsorption/desorption method, the adsorption isotherms of the carbon spheres indicate the presence of mesoporosity (Figure 2a). The corresponding mesopore size distribution is calculated using the Barrett–Joyner–Halenda (BJH) method from the adsorption branch reveals uniform pores centered at approximately 1.3; 2.8; 3.8; 4.7 and 5.7 nm as shown inset of Figure 2a.

The Brunauer, Emmet, and Teller (BET) method has been used to measure of the surface area of our final product. The total specific surface area of mesoporous carbon spheres is 666.8 m^2^/g. It can be attributed to the cavity in the carbon spheres. The powder X-ray diffraction (XRD) pattern shows mesoporous silica, mesoporous silica with CVD deposited carbon and mesoporous carbon spheres (Figure 2)b. The sharp peaks at 2.3; 4.18; 4.8; 6.3^o^ in XRD pattern of the starting template (green line) shows the mesoporous silca structure with empty channels. Peaks at 2.8 and 5.7^o^ in XRD spectrum of the sample after CVD (blue line) are assigned to a mesoporous silica filled with carbon, the strongest peak shifted from 2.3 to 2.8^o^ also indicated the blockage of mesoporous channels with carbon. The overlapped peaks at 22 and 24.9^o^ belong to silica and carbon, respectively. The black line corresponding to the XRD of mesoporous carbon spherical spheres exhibits the strong peak at 24.9^o^ ascribed to graphitic carbon, which is corresponding to Raman spectra. However, the peak at 22^o^ disappeared, which means that silica has been completely removed.

**Figure 3:**
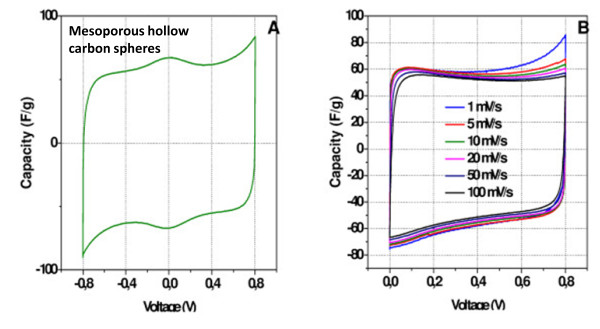
**Cyclic voltammetry curves.** (**a**) Cyclic voltammetry curves of mesoporous hollow carbon spheres in a voltage range from −0.8 to 0.8 V and (**b**) in the range from 0 to 0.8 V at different scan rates

The electrochemical performance of mesoporous carbon spheres are shown in Figure 3. The couple of huge redox peaks are observed in voltammetry curves obtained in the wide voltage range measurement (Figure 3)a. This allows us to assume that the total capacity is a complex of the charging of the electrical double-layer and the pseudo-faradic contribution of the surface functionalities. Because the peaks are located at around 0 V, the pseudocapacity can be generally attributed to the quinone/hydroquinone transformation [13]. For the standard potential window (0–0.8 V), the voltammogram exhibits a quasi rectangular shape in the wide spread of scan rates (Figure 3b). The specific capacity of the carbon spheres estimated from galvanostatic experiment at 0.2 A g−^1^ is 59 F/g. The value is only slightly depended on the current load and even in a low range of current load the carbon spheres show stable behavior.

## Conclusions

Mesoporous carbon spherical spheres were obtained from structured mesoporous silica sphere using chemical vapor deposition (CVD) with ethylene as a carbon feedstock. This procedure does not require additional step to decompose surfactant into carbon. The surface area carbon spheres is large and consists of 666.8 m^2^/g. The electrochemical properties of the carbon spheres, such as capacity of performance at high current load caused that the carbon nanomaterials could be used for supercapacitor applications.

## Competing interests

The authors declare that they have no competing interests.

## Authors' contributions

XC carried out CVD process. KW performed Raman spectroscopy analysis and N_2_ sorption and desorption measurements. KK and JM measured the electrochemical properties of the sample. RK and EM supervised and performed TEM analysis. All authors read and approved the final manuscript.
